# Domestic Violence Between Childhood Incest and Re-victimization: A Study Among Anti-violence Centers in Italy

**DOI:** 10.3389/fpsyg.2018.02377

**Published:** 2018-11-28

**Authors:** Ines Testoni, Chiara Mariani, Adriano Zamperini

**Affiliations:** ^1^FISPPA Department, University of Padova, Padua, Italy; ^2^Emili Sagol Creative Arts Therapies Research Center, University of Haifa, Haifa, Israel

**Keywords:** childhood incest, children sexual abuse (CSA), re-victimization gender-based violence, domestic violence, de-pathologization

## Abstract

The study focuses on a specific problem in the area of child sexual abuse (CSA), which is still under-researched: the relationship between incest and adult female re-victimization treatment within the ambit of domestic violence in Italian centers. About 112 anti-violence centers were contacted, but only 13 participated and only 16 psychologists were interviewed to reconstruct the biographies of 32 victims. The study aimed to examine if and how the service centers recognized and dealt with the problem of re-victimization among survivors based on psychologists’ narrations. Findings showed that the description of perpetrators revealed not only sexual abuse was perpetrated, but also psychological and physical abuse. About half of mothers did not come to their daughters’ aid and those who cooperated with the abusers had mostly suffered from CSA at a time in their life. Only three mothers did help their daughters in contacting the anti-violence centers. However, most of the service centers were not concerned with the relationship between incest and domestic re-victimization, while those who considered the problem, dealt with it only on the practitioner–patient level. In addition, despite the psychologists used professional and empathetic language, they disclosed their high emotional involvement and a genuine bewilderment. A discussion on the need to standardize the psychotherapeutic support given to these re-victimized women was presented, with a critique to the un-discriminated de-pathologization approach adopted by almost all anti-violence centers. In particular, we wanted to underline the fact that, although, this approach is useful in treating victims who were not abused during infancy, it could be insufficient for women who suffered from incest.

## Introduction

Numerous studies on violence against women have been conducted, but there is scarcity of information on the number of women who were sexually abused during their childhood and adolescence. Indeed, it is like a tip of the iceberg in which only the smallest portion emerges while a large run quantifiable part remains unreported (Romito, 2008). Many factors might be responsible: inadequate communication skills to recognize, describe, or remember the abuse, and the social inability to manage all reports of suspected child sexual abuse (CSA) as well as collect and report data (Department of Health and Human Services Administration on Children Youth and Families [DHHSACYF], 2002; Krug et al., 2002). However, estimated findings suggest that one-third of women have experienced sexual abuse while growing up (World Health Organization [WHO], 2017) and particularly during their childhood (Elliott and Briere, 1995). Many surveys have demonstrated how CSA determines adult re-victimization tendency, but very limited research investigated the inverse ratio, that is, the intersection between re-victimization in adulthood and CSA. The importance of this consideration was made evident by a study of the Office for National Statistic for England and Wales (2017), which showed that more than half of adults who were molested during childhood experienced sexual abuse in later life and, in particular, suffered from domestic violence. Actually, CSA is a predictor of adult psychopathology, bringing about anxiety, fear and panic, personality disorders and psychiatric or psychological symptoms, sexualized behavior, sexual addiction or dissatisfaction or sexual maladjustment and disturbance, alcoholism and substance abuse, depression, dissociation, personality and eating disorders, feelings of isolation and stigma, low self-esteem, difficulty in trusting others, self-destructive behavior and suicide (Alexander et al., 1998; Turell and Armsworth, 2000; Glover-Graf and Janikowski, 2001; Dube et al., 2005; Finkelhor et al., 2009; Fortier et al., 2009; Fergusson et al., 2013; Greenfield, 2014; Unlu and Cakaloz, 2016; Hannan et al., 2017; Papalia et al., 2017).

In this area, the entity of incest, which still insidiously and persistently plagues childhood worldwide, is still underestimated as well (Argentieri, 2005; Studer et al., 2011; Middleton, 2013; Pepper, 2014; Aylwin and Reddon, 2017) because of difficulties related to moral/cultural censorship and shame (Pettersen, 2013) or based on the fact that the definition of CSA is quite heterogeneous (Romito, 2008). This type of CSA is the highest form of sexual offense against children and adolescents because they are biological or socio-legally related to the perpetrator (Goodwin, 1985; Greydanus and Merrick, 2017; Pullman et al., 2017; Seto, 2018). In a study by Studer et al. (2011), even though incest is generally considered as a universal taboo and some forms of sanction against sexual abuse by caregivers with children virtually exists in every society; the practical application of this radical prohibition is far from actualized. This significant gap has led to a host of problems in public perception, judicial understanding of offenders, and lastly among treatment providers themselves, while the public has developed a widespread perception that such offenders and their victims cannot be treated (Studer et al., 2011).

Despite the wide spread censorship and the social removal, some studies confirmed the extreme severity of this specific form of violence and its enormous harmfulness. The research conducted by Beard et al. (2017) on 74 victims of father–daughter incest (FDI), compared to 355 controls who were victims of CSA by an adult male other than their father, showed many significant differences between the two groups. It attributed more dangerous effects peculiar to FDI, among which there was a more significant tendency toward re-victimization (Maltz and Holman, 1987; Westerlund, 1992; Sandberg et al., 1994; Messman and Long, 1996). A systematic review has been recently realized by Seto (2018), who widely described the specific nature of this kind of violence and its psychological effects, which appear to be more serious and dangerous than CSA.

Although, it is generally well-accepted that women with a history of childhood sexual abuse are more likely than women without such a history to experience adult violence, research, and dedicated intervention in the field of re-victimization and incest is still needed. In our opinion, the understatement of the latent pathological effects of trauma in battered women is fundamentally due to two factors: on the one hand, to the spread of social censorship because of the generalized disgust that incest elicits, so that people unconsciously do not detect this possible circumstance. On the other hand, with respect to the intervention on battered women, a particular ideological dynamic could be responsible for that underestimation. Our experience acquired from a Daphne Project (Testoni et al., 2013; Bucutǎ et al., 2018), which involved social services in six European countries, we could observe that some sociopolitical points of view have widely shaped the policies and the strategies aimed at supporting battered women. In particular, the idea that it is necessary to de-patologize those victims has been widely spread throughout European and Italian anti-violence services, hugely aimed at improving female agency and at translating the role of victim into that of survivor. This instance of empowerment shadows that of the early feminist critics (Browne, 1993), which stated that almost all the theories that pathologize battered women ignore the power imbalance in relationship abuse, and more or less latently imply that the victim is in some way responsible for the abuse. This idea is perfectly summarized by Lawson (2012), who explains how sociological theories seek to clarify how violent relationships are a function of stereotyped cultural structures rather than individual pathology. The success of this idea is based on the fact that psychotherapists have low competencies on gender based-violence and adapt the survivor’s instances to their narrow interpretative system. Indeed, as reported by Seeley and Plunkett (2002), the psychotherapeutic treatments mostly focused on anxiety and depression for months, without reference to the real traumatic condition of being battered (Lawson, 2012). In particular, the review criticizes the major assumptions (ecological theory, exchange/social control theory, cycle of violence, learned helplessness, system theory, resource theory, and the subculture-of-violence theory) because they are unable to recognize the fundamental influence of culture on gender-based domination of women by men. All these might be due to the lack of a specific psychotherapeutic intersectional approach focused on the treatment of abused women (European Commission [EC], 2009). This tendency has created an important gap in giving an adequate psychological answer to help-seeking women. Since research has already showed that the improvement of these studies could be useful to prevent re-victimization, thanks to specific treatments (Unlu and Cakaloz, 2016; Seto, 2018). In our study, we wanted to check if the psychological effects of incest were considered by Italian centers supporting battered women, in order to acquire wider competence on the concrete strategies utilized in managing this problem. We wanted to investigate whether and how the Italian anti-violence services recognized and treated the problem of the re-victimization of survivors who had earlier suffered from incest, asking psychologists questions about their knowledge of the cases they treated in the past and the characteristics of the narratives brought by the survivors.

## Materials and Methods

This study pertains to the field of qualitative research in psychology (Camic et al., 2003), adopting the qualitative-phenomenological approach considered in the literature to be the most reliable for investigating people’s life experiences and their interpretation of meanings (Denzin, 1995; Gill, 2014; Pietkiewicz and Smith, 2014; Testoni et al., 2018a,b). The combination of the emic view of the participants and the interpretative etic view of the researchers helps the understanding of cultural issues on health and practicalissues (Sanjek, 2000; Oliffe and Bottorff, 2006; Testoni et al., 2018c). Following the CORE-Q check-list (Tong et al., 2007), our analysis is theory-driven, framing interpretations within the studies on re-victimization of battered women who were victims of incest on the basis of literature on gender-based violence (Testoni, 2012). The object of the qualitative analysis was the interpretative repertory on incest and re-victimization, which is a system of descriptions used to characterize and value narrations considered as the gleaming of wider symbolic systems capable of showing the relationships between the two dimensions.

### Participants and Instrument

A total of 112 anti-violence services were contacted, of which 69 did not participate in the research without explaining why they were not interested; 27 declared that they had never encountered cases of incest, 3 declared that they were not interested in this kind of research because they did not treat incest, and 13 provided testimonies. Sixteen psychologists [all female, volunteers or professionals (50%), aged 30–60, average age of 40] were interviewed. They described 32 cases (30 females, 2 males) treated in their work. The structure of the narratives presented a trace of sequential topics, although, they were always presented with great flexibility. A specific questioning route was developed to support conversation, focused on the biographies of survivors (“Please, could you describe the cases of incest that you have dealt with in the past or that you have been dealing with now in your work at the center?”; “How did you hear about the incest?”; “How did you manage this case?”); the kind of incest (“Can you please tell me who and how the perpetrator is or was?”); the family where the incestwas perpetrated and the re-victimization (“Can you describe the origin of the survivors, where the incest happened? Did the survivor describe her life in the incestuous family?”).

**FIGURE 1:**
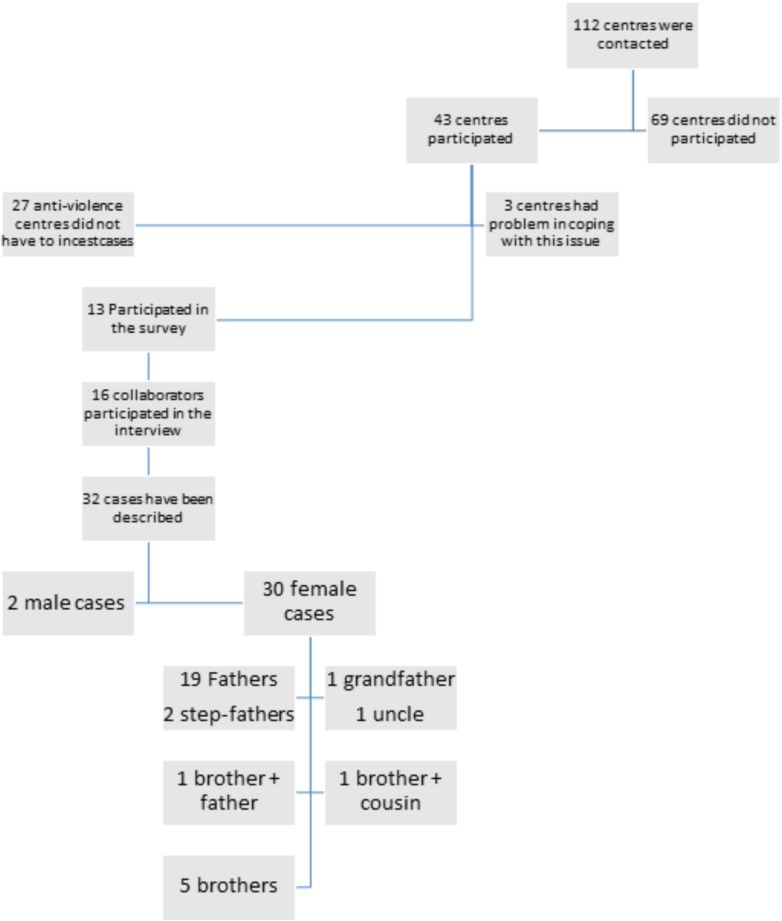
Flow chart describing the phases of the study.

The research followed APA Ethical Principles of Psychologists and Code of Conduct and the principles of the Declaration of Helsinki. After having widely informed participants on the aims and methodology of the study, we obtained the written signed consent form from all participants, as requested by the Code of ethics for the psychology profession – text was approved by the National Council of the Order of Psychologists, Law no. 56/89, revised in December 2006. This code is mandatory and well-known by all psychologists and does not explicitly require that research among psychologists should be submitted to ethics committees. This rule is applicable at both national and institutional level (Figure 1).

### Data Analysis

The interviews were transcribed and analyzed using the framework method for thematic qualitative analysis, which allows sources to be examinedin terms of their principal concepts or themes (Marshall and Rossman, 1999). Two researchers used this approach, which is particularly appropriate, especially in health care and during research of sensitive problems (Pope et al., 2000). The process was pivoted on both prior categories and categories which only became clear as analysis proceeded (Testoni et al., 2017). The former were the basic “pre-fabricated themes” (incest, initial assessment, family of the victims, kind of support …), from which the latter emerged as unexpected topics. The process was divided into six main phases: preparatory organization; coding data; generation of categories or themes; testing emerging themes; searching for alternative explanations; writing up the report (Marshall and Rossman, 1999). Thematic analysis was performed with Atlas.ti, whose outcome results in network graphs, describing logical relationships between concepts and categories identified by researchers (Testoni et al., 2016).

## Results

From the codification of the text, four areas of thematic prevalence have been identified: the relationship between offender/victim and mother/victim, the consequences of the abuse, as well as the personal experiences of the practitioners.

### The First Area of Thematic Prevalence: The Relationships Between Victims and Offenders

**FIGURE 2:**
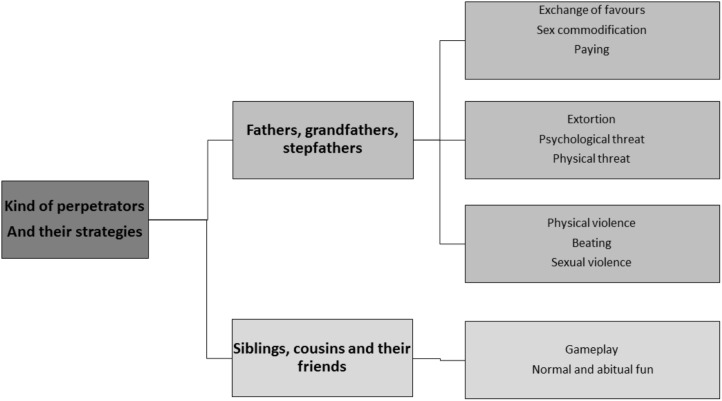
The offenders and their strategies.

In the analysis of the relationship between the victim and the offender, psychologists described the role that the perpetrator of the incest played in the family and the kind of incest. It emerged that in 19 cases the offenders were fathers, in 5 brothers, in 3 stepfathers, in 1 grandfather, in 1 uncle, in 1 both father and brother, in 1 both brother and cousin at the same time, and in 1 both brother and a friend (Figure 2).

Sexual abuse went together with physical aggression, cruelties and violence in every aspect of life accompanied by blackmail and torture. In particular, FDI dynamics were, on the one hand, managed in the form of prostitution by purchasing sexual relationship with daughters whom the fathers paid with money and favors: “If, for example, she needed to buy something, she had to undergo sexual abuse. This type of arrangement led her to believe she had an agreement to some extent with her father and, in particular, be in a favorable position among the siblings. She felt somehow that something was wrong, and guilt for what happened in her childhood and she was unable to manage this memory.” “Often, these victims felt they were co-responsible for the sexual relationship with their father because they had economic advantages. As a result, their fathers taught them to exchange sex for money and gifts.” These descriptions stressed how a father taught his daughter that it was possible to fulfill her desires only by providing him sexual favors. Sex was presented to be the unique means of exchange for the satisfation of any need. Even the simplest needs, such as the purchase of school supplies or standard daily clothing, were intercepted by blackmail strategies. What perhaps was the worse shakedown which consisted in the limitation of relational freedom with peers and other people: “Possessiveness and a distorted form of jealousy or even the fear that someone would know or understand the problem were the triggers that activated in the father the request for settlement by the sexual pledge in exchange for the possibility of having relationships out of family with friends.” In such a situation, the victims could not cultivate a regular relationship or an authentic confrontation with their peers and could not safely live a sentimental life with the first sexual experiences.

On the other hand, when victims tried to escape sexual abuse, they were always subjected to torture and punishment: “They suffered from stalking, threats through blackmails, punishment like being refused money for snacks, clothing, books and school materials. The most frequent punishment was segregation at home, but there was also physical torture, such as burning and beatings.” “They were subjected to sexual violence from which they could not free themselves and the easiest way to endure was to suffer, waiting for it to end.”

On the contrary, the incestuous relationship with brothers had a different connotation and the most frequent metaphor was that of “games”: “She believed that sexual relationships between siblings are quite common ‘because they are things that everyone enjoy’.” “She was convinced that normal relationships between siblings may include sexual experiences and that the abuse perpetrated by brothers could be acceptable.” “They were not able to discern whether it was acceptable or not, whether they were consenting or not.” “She found it a bit strange, she felt confused because she could not understand since she had been consenting or refusing, violated or involved.” In a case, the abuse continued after the death of the father: “A woman in her thirties abused by her father was still suffering from abuse by her brother. Father and brother had already abused her when she was a child and an adolescent; after the death of the father, she continued to suffer abuse from the brother” (Figure 2).

In half of the cases, victims kept the secret till adulthood: “They did not disclose to anyone because they thought that their experience was not conceivable and that they were not able to immediately tell others about the abuse.” “By concealing the problem at a time when violence was reoccurring, victims believed that somehow the situation would go away. In almost all cases, the experience of sexual abuse was later revealed in adulthood to friends, relatives or partners.” “In one case, the abuse was revealed for the first time after 58 years.” “They could freely speak about the abuse once the offender left the family because of separation.” “She confessed the problem only when he left.”

### The Second Area of Thematic Prevalence: The Role of the Mother

**FIGURE 3:**
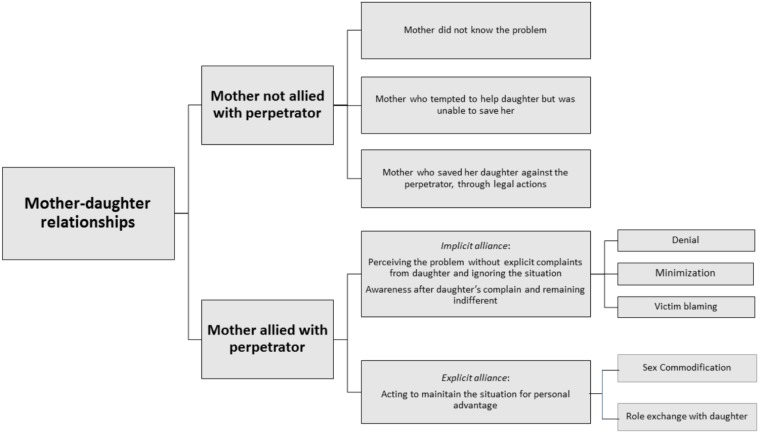
Reactions of mothers.

In none of the cases described did the mothers actively play the role of sexual offenders. The two males who tangentially appeared in the narrations suffered from continuous sexual violence from their fathers. In the description of the female cases, particular attention was paid to the mother’s position and essentially two roles emerged: the mother who protected her daughter and the mother who did not (Figure 3).

#### The Mother Who Protected the Daughter

From the narrations, only three cases appeared with the reporting of the perpetrator, thanks to the mothers’ help: “They accused him during the process and were able to face him directly in the eye.” On those occasions, the perpetrators were removed from the family: “In one of the very few cases, seen in so many years of work, he was later deprived of parental authority. He was removed, charged, condemned and put in prison.” Only these mothers were reported by the practitioners to be unaware of the abuse suffered by their daughters. In the first case: “It seemed that she never noticed it, at least, that was what she said. Honestly, I don’t know if this was true or false. However, she was a patient of the center because her husband battered her and so we could also help her daughter.” In the second case, the mother asked to be helped by the center with respect to the daughter: “She couldn’t believe what happened and she was shocked when her daughter told her everything. She felt lost. It was very difficult for her to accept that her husband was a sexual offender against her daughter, but after the initial shock, she understood the situation and denounced her husband. She was able to support her daughter and help her through all the phases after denouncing her husband. She tried to build a protected environment. However, she had to create a therapeutic path for her to overcome the trauma.” The third case was on a victim of domestic violence, who “suffered from physical violence for a long time, but when she became aware that he was sexually abusing her daughter, she asked to be placed in a separate house and denounced the perpetrator. The sexual abuse and incest gave her the courage to rebel against her husband.”

#### The Mother Who Did Not Protect Her Daughter

Most of the second types of cases pertained to the biographies of re-victimized women who asked to be helped by the centers because they were victims of violence by their husbands. During their childhood, they had also suffered from sexual abuses by their fathers, brothers, grandfathers, and cousins without any support or help from their mothers. In the opinion of the victims, most of the mothers knew what was happening; however, this was not always certain. The most common statements were: “She said that her mother knew everything and did not protect her”; “Victims tried to ask for help from their mothers, disclosing their situation, but it was in futility.” Three main maternal forms of reaction to the daughter’s disclosure appeared in the narrations. In the first modality, the mothers denied that the abuse actually took place: “The mother seemed not to have believed a single word by the daughter.” “Sometimes, mothers ignored or dismissed the daughter’s confessions.” In the second modality, mothers minimized the problem: “I remember a victim who tried to understand what was happening. She asked her mother for help, but her response was ‘After all, what did he ever do to you?’ She tried to ask friends for help, but she only managed to escape violence by leaving home.” In the cases of incest with brothers: “The mother accused their daughter of being envious of her brother and explained away the problem, considering it all as a banal slander.” “Often, mothers did not listen or pay attention to the stories their daughters told them.” “There was a case in which the mother, in the name of religion, told her daughter to respect and forgive the father. She was evidently unable to manage the situation.”

The third modality was characteristic of mothers being evidently collusive with their husbands: “The pact between parents was that the father could sexually abuse their daughter in exchange for favors and money for the mother. Of course, the requests for help by the victim were ignored and she was supposed to accept the abuse by the father.” In another case, “The father would openly touch the intimate parts of both the wife and the daughter. When the second one asked for help, the mother answered that the hassle would have taken a while and that it would be over quickly, so the best thing to do was to be patient. This answer showed that there was a solid alliance between the parents.” “In another case, the daughter was told that she could not do anything to stop the situation. She had to assume the role of the mother, in all and for all, so she had to be sexually abused to take care of the other brothers.” “In another case, the mother was not able to protect the daughter because she was like a sister; instead of assuming a protective role, she was an impotent friend who did not know what to do. The mother went into depression and the daughter felt lost.”

### The Third Area of Thematic Prevalence: Consequences of the Abuse and Re-victimization

The psychologists’ narrations widely emphasized how incest has seriously compromised the normal development of victims’ personality and agency. “She learnt to be passive, unable to defend herself, unable to set limits.” “These victims, in adulthood after having suffered from incest, maintain infantile traits.” “She behaves like a girl, but she is in her 40s, and she has an incredibly infantile aspect, truly with the physical traits of a girl.” “She is a girl. Looking at her, you cannot guess her age, she has some very infantile traits, extremely infantile.” This inhibition could have been derived from the psychodynamic effect of the self-blame, related to the idea that the sexual relationships with the father produced some convenient advantages. Going by the opinion of our participants, this beliefs undermined the victim’s self-esteem and sense of personal “dignity” and “integrity.” This “devastating interference” led to a limitation in the normal psycho-affective and sexual development. With regard to the re-victimization, practictioners suggested that the latent self-blame related to the sexual relationships with father could have undermined the ability to select and develop friendships, influencing the wrong choice of violent partner in adulthood: “She was not able to measure herself against her own affective needs in her relationship with her peers and, in adulthood, she was not capable of meeting her own needs with a good partner.” As time went on, from a clinical point of view, a vast range of disorders emerged from the stories, testifying to the severe symptoms of post-traumatic syndrome: “Eating disorders, obsessive-compulsive behavior, and increased need for control of one’s life or of one’s body, hypochondria.” “They suffered from panic attacks and couldn’t study or take exams,” “She suffered from anxiety disorders and depression that she treated with drugs.” “She felt distrustful and insecure in relationships with others, especially with men.” “She had many sexual and gynecological problems.” “In her relationships with partners, she encountered several sexual difficulties.” “She vomits and experience sleep disturbances.” “When she was a child, she and her friends behaved sexually with each other as well as played sexual games. During recreations she would put her hands in the panties of other children.” In most cases, there were feelings of shame and guilt: “Above all, they were ashamed of this experience, and were not anxious to speak about it. They preferred to conceal everything.” “They felt guilt and shame and a deep sense of loneliness.”

Psychologists were evidently able to recognize the most important effects of incest. Nevertheless, our impression was that the participants did not refer to precise models of interpretation of the consequences of this type of abuse. Their empathic competence was not reinforced by their reference to a specific literature that could support their deep insights. Certainly, the cause of this deficiency is not to be attributed to them, but rather to an evident deficiency in research in this field and therefore, to an absence of empirically validated models in the psychological clinical practice that can support their work from diagnosis to measurement of the effectiveness of the intervention and the follow up.

### The Fourth Area of Thematic Prevalence: The Feels and Involvement of the Psychologists

Participants in our research had always used a language appropriate to the role played by the anti-violence service. However, despite their consolidated professionalism concerning domestic violence, they used some expressions that unveiled their stressful emotional involvement. In particular, their surprise with regard to the problem of incest. Many cases were described as horrible, unbelievable and unbearable: “It was a chilling story.” “It was a shocking situation.” “It was hallucinating.” “It is impossible that all this can happen now, in this epoch, in our cities!” “It was similar to a horror tale.” “Sincerely, I had never imagined that such situations could exist.” In other narrations, psychologists said: “I have had difficulty listening to the victims’ story despite many years of work at the center. And this was even after so many years of work, and stories you know, I was petrified, I told her and I was only able to say that I was close to her in her pain.” “I have trouble talking about it; it is difficult to describe what I listened to.” “It is difficult to maintain professional distances from these narrations, especially when the victims describe the torture they suffered. You have to think that the perpetrator has psychiatric problems, that he is pathologically affected by sadism, to avoid hating him. It is not easy, really; it is disgusting and quite impossible not to define such perpetrators as beasts.” “Really, it is difficult to believe that she was raped by her father.”“I cannot bear the condition of these victims who seem similar to puppies, wounded, and unable to defend themselves.” On the one hand, all the expressions used to describe the personal experience in facing the victim’s effects of incest have highlighted the empathetic involvement of the operators, and therefore their ability to understand the suffering of the victims. This was undoubtedly their strongest instrument in dealing with the problem, but on the other hand, all this revealed the lack of appropriate clinical instruments, such as assessment, statistics inherent to the problem, relationships between characteristics of incest and perpetrators and pathological or psychosocial consequences. In fact, the descriptions showed that the discovery of incest was made, thanks to the narratives of the victims who asked for help at the center. Furthermore, the concept of “re-victimization” was only incidentally utilized, in order to show the continuity of the violence suffered, due to the passivity learned by the victim during childhood, for the paternal abuse. The relationships between incest and re-victimization were not described analytically and all the descriptions were in the past. No surveys or investigation focusing on the problem were realized to explain the current victimization and CSA and incest. However, since there are no precise approach in the psychological field that orients both the diagnostic survey and the intervention strategies with respect to incest and re-victimization, none psycho-diagnostic assessment could be systematically carried out in order to identify the factors involved in the problem. Unfortunately, this result runs in parallel with our concrete experience with many Italian anti-violence centers, where we could see that no clear assessment and clinical instrument were yet to be utilized in treating the victims’ post-traumatic effects of violence.

## Discussion

Our research was aimed to highlight whether and how Italian anti-violence services managed the incest experiences suffered from help seeking women, by analyzing the psychologists’ narrations of the cases and their possible representations of the problem of re-victimization. In this discussion, we underlined the fact that our participants were the only ones faced with the question, unlike all others working in centers that have not even considered our proposal to address the problem. However, our perception was that there is an important need for assessing this specific problem and furthermore for the treatment of re-victimization pertaining to domestic violence in adults as outcome of incestuous CSA experiences.

In Italy, the socio-constructionist de-pathologizing approach, which promotes victims’ autonomy to decide their own life because they have the competency to do that (Teles, 2008), has emerged as the most important perspective, of which we wanted to check how the centers managed the problem. No matter how much we agree with the pivotal concept that illustrates the sexist role of gender stereotypes (Dua, 2006; Graham-Kevan, 2007; Sharf, 2011), as already discussed by literature, we assumed that such an approach is particularly effective when assisting women who have issues within a higher level of functioning in contemporary society (Ballou et al., 2008; Sharf, 2011). Indeed, despite this advantage, it is limited by its focus on social change, and attributing blame to external sources for women’s problems can result in underestimating the individual’s contribution to their own problems (Dua, 2006; Jordan, 2008). Specifically, it ignores the effects of the reiterated sexual trauma in childhood.

First of all, it is important to highlight that, with regard to all the research, we adopted the Pro Woman Line (PWL) (Robinson Hancock, 1972; Hanisch, 2006), which is the pivot of the early black feminist perspective, whose point of view was that any female behavioral expression resulted from subservience of women to men (McIntyre, 1981; Wattenberg, 1985). From this point of view, sexual abuse within the family is a source of female victimization resulting from a patriarchal culture, that is, male control over women and children (Liebman Jacobs, 1990; Ehrmin, 1996). In particular, we shared the idea about the classical psychological bias of “mother blaming,” establishing that in the case of FDI, the mother possesses certain personality traits and sexual inadequacies that allegedly provoke the incest because she is not fulfilling her sexual role, is undoubtedly sexist. However, the fundamental assumption of PWL helped us not to judge any women involved in our research; at the same time, we tried to recognize the roles of all the actors involved in the incest descriptions. In particular, males evidently appeared as violent perpetrators who not only abused their daughters, sisters, and cousins, they furthermore used their power to perpetrate psychological and physical violence. Nevertheless, most mothers did not help their daughters. It was in only three biographies we found the mothers defending their children and contacted the anti-violence services. In the other circumstances, mothers were not able to rescue the victims from violence, and even, in about half the situations described, they were collusive with the offenders. These conditions were really similar to those described in literature, which evidenced that re-victimization is associated with a history of incest, CSA, exposure to dangerous contexts and to receiving a greater proportion of negative reactions when disclosing assault (Relyea and Ullman, 2017). Furthermore, as Bennett (1990) already hypothesized, mothers’ limited capacity to protect their daughters run in parallel with their defensive disbelief and evasion of the incestuous relationship because of their longstanding separation disorder and their low level of self-esteem, which limited their ways of dealing with anger in relationships. Their lack of urgency and their incapability to react aggressively against their husband allowed him to abuse their daughter sexually. Furthermore, independent of mothers’ behavior, the violated children described by our practitioners had been re-victimized in adulthood by their husbands and companions (in one case by the brother), and that was the reason they asked for help from the centers. This confirms that, when the problem is unchecked, women who suffered from incest in their childhood may be re-victimized in adulthood. Furthermore, the important research of Lev-Wiesel (2006) aimed to understand why the dynamics of sexual abuse is perpetuated across successive generations. Her analysis on 24 mothers who were survivors of incest and whose children were victims of incest discovered four types of motherhood: the unaware mother, characterized by a complete lack of cognitive knowledge of the sexual abuse; the unwitting accomplice, characterized by latent cooperation with her husband; the enabler, characterized by encouraging her spouse in the raping of her daughter; and the common fate mother, characterized by sharing a common fate with her daughters. This study unveils one important factor that can determine the intergenerational continuance of FDI and illuminates one aspect of the relationships between mother and daughter, which should be better recognized to stop the possible victimization of new female generations.

With regard to the area inherent to the competencies of anti-violence services on this issue, we remained astonished by the fact that only 13 centers over 135 of them actively participated in the research and that only their practitioners were able to describe cases of incest. Maybe the initial reason the centers did not participate in the survey was related to the fact that in the last decade, in Italy, many studies have been realized involving them, and then our proposal might have appeared insufficiently appealing. However, even though most of the centers did not explain why they were not interested in the study, those who answered chiefly asserted that their patients were not re-victimized survivors of incest and CSA. This result may partially confirm that the problem is still underestimated and the relationships between incest and re-victimization have probably not been sufficiently surveyed. Indeed, most of the Italian anti-violence services are guided by the de-pathologizing approach, which is a non-victim-blaming therapy, offering some fundamental advantages, among which is the enhancement of values that support a patients’ self-esteem and growth through empowerment (Jones-Smith, 2011). Nonetheless, despite this important cultural pivot, there remains little scientific evidence to suggest that the effectiveness of this approach is better than the psychological ones. In the light of these conjectures, the European Commission (European Commission [EC], 2009) recommendations, which denounced the fact that too many generalist practitioners lack the specialized knowledge, competence and confidence necessary to counsel victims of domestic violence, are particularly significant. In the EC’s opinion, this kind of survivors need skilled interventions enabled by dedicated courses that are developed from evidence-based methods.

Thereafter, we emphasized the importance of psychological research in this field, aimed at improving the discovery of relationships between CSA, incest and domestic violence re-victimization. Precisely, if the early studies on CSA and incest focused on sexual re-victimization, most recent researches are centered on all other forms of violence, among which are domestic and intimate ones (Courtois, 2012; Relyea and Ullman, 2017). In particular, we hypothesized that, since sexism is still a reality in the Italian society, maybe specific behavior of CSA and incest victims can expose them to re-victimization, among which are talking about sex and their sexual experiences (Russell, 1986; Stroebel et al., 2012), or being disposed to engage in sex for money (Stroebel et al., 2012), or having sexual difficulties (Stroebel et al., 2013; O’Keefe et al., 2014). Indeed, such an enhancement could be a crucial form of prevention of re-victimization, in consideration of the fact that re-victimization is related to submissive behavior, interpersonal dependency, self-blame and low self-efficacy that characterize the adult personality of women who suffered from child CSA and incest trauma (Brunngraber, 1986; Herman et al., 1986).

It appears that, although, psychology has already provided useful instruments to assess and treat incest survivors (Fassler et al., 2005; Welldon, 2005; Courtois, 2010; Dinnen and Cook, 2011; Palmer, 2011; McElroy et al., 2016; Seto, 2018), there is still a great need to spread such competencies in social health and anti-violence service centers. Certainly, it is difficult to assess the biographical data of survivors of domestic violence and to especially check the area of incest. This difficult early phase of the intervention should be considered by the anti-violence service centers, in order to recognize the most important psychosocial factors that determine victimization and requiring specific characteristics.

## Conclusion

Our research showed that the problem of re-victimization in adulthood for women who suffered from incest during their childhood is still under-surveyed by Italian anti-violence services. Since incest in childhood and adolescence is a traumatic experience that could severely interfere with the normal psychological development of survivors, it is crucial to develop methodologies and surveys to treat this kind of survivors psychologically. Finally, we wanted to discuss a paradoxical problem, which may hamper intervention for victims of domestic violence. Specifically, we wanted to underline that battered women or abused children/adolescents are not pathological because they suffer from violence, that is, they are not necessarily abused because they have some psychopathologies that expose them to a violent intimate relationship. Undoubtedly, they are victims of offenders who can be mentally insane and of society that is culturally and structurally unable to prevent these aggressions. However, after the violence, survivors may suffer from psychological problems as an effect of trauma. Suffering from the effects of trauma is not only a cultural problem, but the anti-violence services should offer the victims a specific form of psychotherapy and not merely a supportive intervention for their empowerment. From this perspective, we wanted to consider whether and how the problem of re-victimization and its relationships with incest trauma had been treated in Italian anti-violence services.

Finally, this study confirmed what the European Commission already underlined, or that there is a relative dearth in previous evaluation studies in many of the countries of the EU that addressed this issue. There is, therefore, a need to understand why anti-violence services do not routinely evaluate the problem or, if they do, why findings from these studies are not widely disseminated. These studies could reinforce the ability and competence of intervention aimed to elaborate trauma, to empower female and child victims of CSA, incest and domestic violence, their re-construction of self-autonomy, and their own decisions regardless of the violence they have experienced.

However, the limitations of this study are numerous. On the one hand, it was very difficult to understand why many of the service centers contacted were unwilling to participate in the survey. On the other hand, it is not clear whether those who answered did not have cases of incest because they surveyed the problem with their patients and they never had cases of re-victimization or whether they simply avoided the issue. At least, the analysis is qualitative, so it does not permit generalizing the results.

## Author Contributions

IT, CM, and AZ contributed equally in writing and reviewing the paper.

## Conflict of Interest Statement

The authors declare that the research was conducted in the absence of any commercial or financial relationships that could be construed as a potential conflict of interest. The reviewer YC and handling Editor declared their shared affiliation.
